# Expression pattern of glucocorticoid receptor α gene and associations with clinicolaboratory features in patients with systemic lupus erythematosus

**DOI:** 10.22088/cjim.14.3.470

**Published:** 2023

**Authors:** Maryam Sahebari, Zahra Rezaieyazdi, Mandana Khodashahi, Houshang Rafatpanah, Hassan Mehrad-Majd, Nayereh Saadati, Masoumeh Salari

**Affiliations:** 1 Rheumatic Diseases Research Center, Mashhad University of Medical Sciences, Mashhad, Iran; 2 Department of Immunology, Faculty of Medicine, Mashhad University of Medical Sciences, Mashhad, Iran; 3 Clinical Research Development Unit, Ghaem Hospital, Mashhad University of Medical Sciences, Mashhad, Iran

**Keywords:** Systemic lupus erythematosus, GRα gene, CRP, Lupus, Glucocorticoid gene receptor.

## Abstract

**Background:**

Glucocorticoid receptor α (GRα) gene is a transcription factor with clinically significant immune-modulating properties in various autoimmune diseases. However, the expression pattern of the GRα gene and associations with clinical features in patients with systemic lupus erythematosus (SLE) is controversial. This study aimed to assess the correlation between the GRα expression and different clinical and laboratory-related parameters in SLE patients.

**Methods:**

A total of 45 women with newly diagnosed SLE and 31 gender and age-matched healthy controls were enrolled in this cross-sectional study. The real-time quantitative PCR (qRT-PCT) method evaluated the differences in GRα expression in peripheral blood mononuclear cells from cases and controls. The correlation between the GRα gene expression levels, clinicolaboratory features, and potential prognostic application was also analyzed.

**Results:**

Compared to the healthy individuals, the GRα gene expression in newly diagnosed SLE patients who did not receive any treatment was numerically reduced, but this reduction did not achieve statistical significance (P=0.87). No significant correlation was also found with the activity and severity of SLE according to SLEDAI2K (P=0.41). The GRα gene expression showed a negative correlation with CRP (P=0.034) and a positive correlation with lupus anticoagulant (P=0.039) levels in SLE. The receiver operating characteristic (ROC) curve analysis indicated that the GRα expression level might be a predictor biomarker for low CRP and positive lupus anticoagulant in SLE, respectively.

**Conclusion:**

This study proposed that expression of the GRα in newly diagnosed lupus patients has no statistically significant difference with healthy age and sex-matched controls. Besides, its expression does not correlate with lupus disease activity according to SLEDAI2k. However, further studies in this area are required.

Systemic lupus erythematosus (SLE), as one of the most well-known autoimmune diseases in women, is an autoimmune disease with diverse clinical presentation, complex pathogenesis, and multiple etiologies (1,2). Despite considerable efforts for finding the best therapeutic strategy for SLE patients, still, glucocorticoids are the most available and lifesaving option for the treatment of SLE (3). In the majority of the cases, the response rate to glucocorticoids is favorable to ameliorate the symptoms of the disease significantly. However, the risk of glucocorticoids resistance could threaten the life of a particular group of patients (1). 

It should be noted that the glucocorticoid-resistance not only could attenuate the therapeutic effects of exogenous glucocorticoids but also induce resistance even to endogenous glucocorticoids (4,5). According to the current therapeutic protocol, higher doses of glucocorticoids are being used in resistant patients, increasing the risk of morbidity due to organ damage, opportunistic infections, osteoporosis, and avascular necrosis (6). Thus far, several mechanisms of resistance to glucocorticoids have been identified; however, alteration in the expression level of glucocorticoid receptors (GRs) might confer resistance phenotype in lupus (7-10). GRs are members of nuclear hormone receptors that, in response to glucocorticoids, translocate from the cytosol into the nucleus. They could orchestrate a wide range of physiological functions by regulating the expression of different downstream genes (7, 11). Two isoforms of GRs have been so far identified, which among them, GRα is the dominant receptor form. A few hypotheses proposed that the expression of GRα in SLE patients and thereby accelerate the progression of the disease. Although the genetic abnormalities in the GRα gene encoding, such as polymorphisms. Glucocorticoids play an essential role in inhibiting inflammatory cascades and cytokines. Therefore, the use of glucocorticoids is the first line of treatment in many autoimmune diseases. During lupus, it is expected that the expression of the receptor gene, followed by the expression of glucocorticoids at the cellular level, will decrease and cause the onset of inflammation.Whether this hypothesis can be associated with clinical and laboratory symptoms is discussed in this article.(12-14), overexpression of p-glycoprotein (p-GPro) (15,16), the elevation of macrophage migration inhibitory factor (MIF) (17), and overexpression of TLR9 (18) are all the other postulated mechanisms that could indirectly reduce the expression of the GRα in lupus. In a study conducted on 60 SLE patients, it became evident that those patients with a severe type of the disease had higher expression of p-Gpro than their healthy counterparts (19), suggesting that the expression of this protein could be associated with the aggressive type of the disease. In another study, it has also been reported that the elevated expression of MIF could accelerate the progression of SLE in the patients through MAPK-dependent suppression of GRα (20). Despite the importance of understanding the mechanisms that lead to the downregulation of the gene encoding this receptor, taking the veil off the roles GRα gene might play in the pathogenesis of the disease could also be beneficial for the management and treatment of SLE. Thus far, the majority of the studies suggest that the downregulation of the GRα gene occurs. Given this and based on the importance of the GRα gene expression in the pathogenesis of the disease, the present study was designed to explore the relationship between the GRα expression level, risk of SLE, disease severity, and related clinical and laboratory features.

## Methods

**Patients: **According to the estimated sample size based on the GR gene expression levels in patients with SLE and corresponding healthy controls in a previously published study (13), With α=0/05, β=0/20 (80% power), at least 43 people are needed in each group. Forty-five women aged more than 18 years with newly diagnosed systemic lupus erythematosus (SLE) referred to Rheumatic Diseases Research Center (RDRC) were enrolled in the present cross-ot5ir sectional study. SLE was diagnosed according to the SLICC-2012 criteria (21). All patients did not receive any medication before the study. Those previously treated with corticosteroids, or those with other autoimmune diseases, overlap syndromes, and endocrine diseases with elevated or decreased expression of endogen glucocorticoids, were excluded from the study. SLEDAI-2k is a scoring system through which SLE patients could be categorized into mild (score < 4), moderate (score =4-10), and severe (score > 10) groups (22). Moreover, 45 healthy women without any familial history of SLE were selected to be the control group. The median age of the patient (34.8 ± 9.9 years) and the control group (37.8 ± 4.1 years) was quite the same (P = 0.074). Blood samples were collected from all included subjects. The sample of 14 healthy individuals was discarded from the study due to insufficient volume or clotting. The number of individuals in the control group was reduced to 31. All participants signed an informed consent form, and the Ethics Committee of Mashhad University of Medical Sciences approved the study protocol (ID number: IR.MUMS.MEDICAL.1397.470).

**Peripheral Blood Mononuclear cell (PBMCs) isolation: **About 3 mL of fresh blood samples in the tube containing EDTA, as an anticoagulant, were mixed with 3 mL of PBS and ficole. After centrifuging at 2500 rpm for 20 minutes, the buffy coat layer was isolated and transferred into a PBS tube. The mixture was centrifuged for 10 min at 2500 rpm, the supernatant was discarded, and the pellets containing peripheral blood mononuclear cells (PBMCs) were dissolved into 0.5 mL of PBS for further evaluation. 

**RNA extraction and the qRT-PCR analysis: **PBMCs were exposed to an RNA extraction reagent named TriPure (Roche, Germany) to extract RNA from all samples. The quantity of the extracted RNAs was measured at the wavelength of 260 nm using NanoDrop 2000 spectrophotometer (Thermo Fisher Scientific, Waltham, MA, USA). Moreover, agarose gel electrophoresis was done to test the quality of RNAs. For each patient, 2μg of extracted RNAs were used to synthesize the complementary DNA (cDNA) using a cDNA synthesis kit (revertAid First StrandcDNA Synthesis Kit (Takara BIO)). The synthesized cDNA, along with the specific primers; (GRα, 5'-TGCCGCTATCGAAAATGTCTT-3' (forward), 5′-GGGTAGGGGTGAGTTGTGGT-3′ (reverse); β2-microglobulin (β2M), 5'-CTTGTCTTTCAGCAAGGACTGG-3' (forward) and 5'-CCACTTAACTATCTTGGGCTGG-3' (reverse)), were then subjected to SYBR green-based real-time quantitative polymerase chain reaction (qRT-PCR) (light cycler, Roche, Germany). The condition for thermal cycling was as follows; initial activation step of 30 s at 95 °C, 40 cycles of a denaturation step (15 s at 95 °C), and a combined annealing/extension step (60 s at 60 °C). An endogenous control was performed by using β2 microglobulin (β2M), the result of GRα expression was normalized to β2M expression, and the relative quantity of the gene of interest was normalized to the relative quantity of β2M and was reported as relative mRNA expression.

**Statistical analysis: **All statistical analyses were performed by IBM SPSS Statistics 25, and a p-value<0.05 was considered statistically significant. The chi-square and t-tests were applied to assess the possible association between GRα expression levels and risk of SLE and related phenotypes. Mann-Whitney test or Spearman correlation analysis was also used to evaluate any association between GRα expression levels and clinical parameters. Moreover, to determine the sensitivity, specificity, and likelihood ratios of GRα gene expression and the levels of CRP and lupus anticoagulant test, receiver operating characteristic analysis (ROC) was performed.

## Results

**Figure 1:**
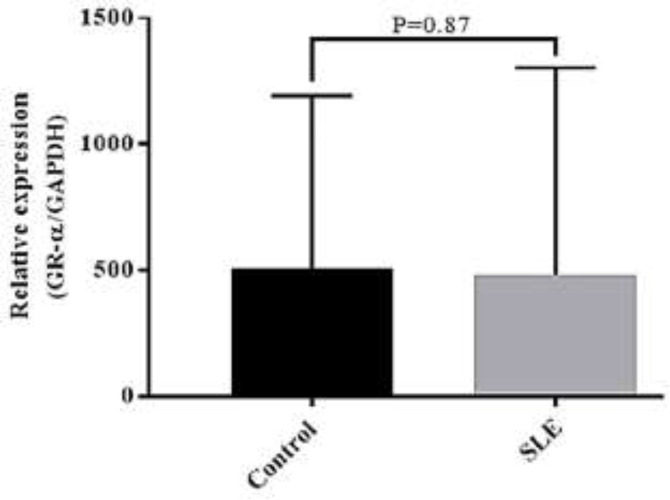
GR-α mRNA expression levels in PBMCs from 45 SLE patients and 31 healthy controls

The expression of GRα gene in patients with systemic lupus erythematosus (SLE). This study chose two groups of SLE patients with the median age of 34.8 ± 9.9 years and the control group with the median age of 37.8 ± 4.1 years old (P = 0.07). Demographic, clinical, and biochemical data of 45 SLE patients and 31 healthy controls are presented in tables 1and 2). Comparing the physical features of both groups, we found that the BMI of the SLE group was significantly lower (P = 0.001). Apart from the physical features, the results of the qRT-PCR analysis revealed that although the expression of GRα was lower in the PBMCs isolated from SLE patients as compared with the control group, this reduction was not statistically significant (P = 0.870) (figure 1). 

**Figure 2:**
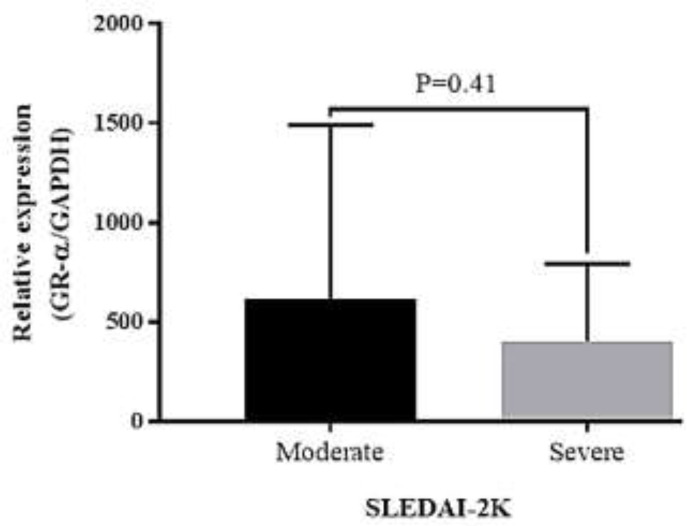
GRα mRNA expression in SLE patients subgroups based on the SLEDAI-2K scores

The correlation between GRα gene expression and the activity and severity of the disease. To evaluate the association of the GRα gene expression with some clinical features in patients, disease activity was divided into mild (score < 4), moderate (score =4-10), and severe (score > 10) based on the SLEDAI-2k scoring system. Twenty-three (51%) patients had the moderate type of the disease, and 22 (49%) patients suffered from the severe type of the disease. Compared with the patients with moderate SLE, those who had a severe type of the disease had non-significant lower expression levels of the GRα gene. Mann-Whitney result also indicated the lack of association between GRα gene expression levels and the activity and severity of the disease (P = 0.414) (figure. 2).

The correlation between GRα gene expression and the clinical laboratory features of SLE patients. As presented in the Table 1, GRα gene expression had no significant correlation with mouth ulcer (P = 0.08), butterfly rash (P = 0.45), hair loss (P = 0.8), fever (P = 0.7), headache (P = 0.76), CVA (P = 0.13), vasculitis (P = 0.87), arthritis (P = 0.96), myositis (P = 0.85), and serositis (P = 0.70). Correlation analysis between GRα gene expression levels and various laboratory parameters of the patients such as proteinuria, hematuria, sterile pyuria, glomerular filtration rate (GFR), ANA, anti-dsDNA, anti-Sm, anti-cardiolipin, anti-β2 glycoprotein, lupus anticoagulant, ESR, leukopenia, lymphopenia, thrombocytopenia, anemia, CRP, C3 andو C4 deficiency was also performed. As shown in table 2 GRα gene expression was inversely correlated with CRP (P = 0.032). Moreover, a significant positive correlation was found regarding lupus anticoagulant (P = 0.039) (table 2). 

**Figure 3:**
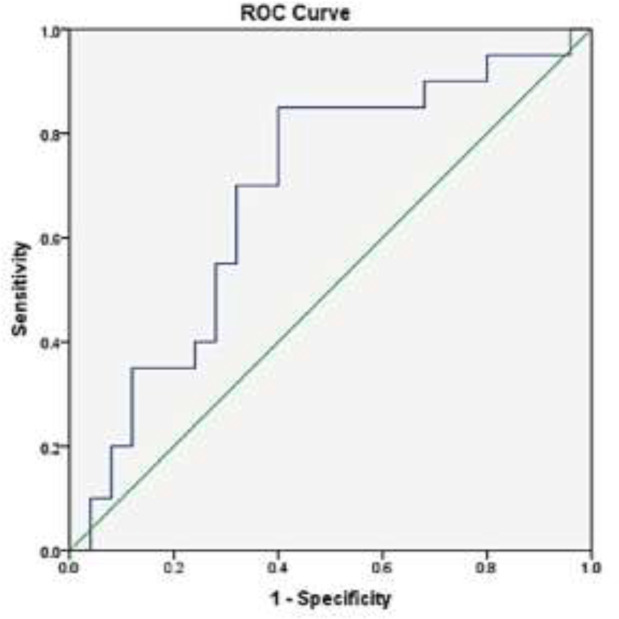
The ROC curve for determining the GR-α expression cutoff value for identifying SLE patients with elevated CRP levels. AUC=0.70; 95% CI: 0.53–0.85, P=0.034

**Figure 4:**
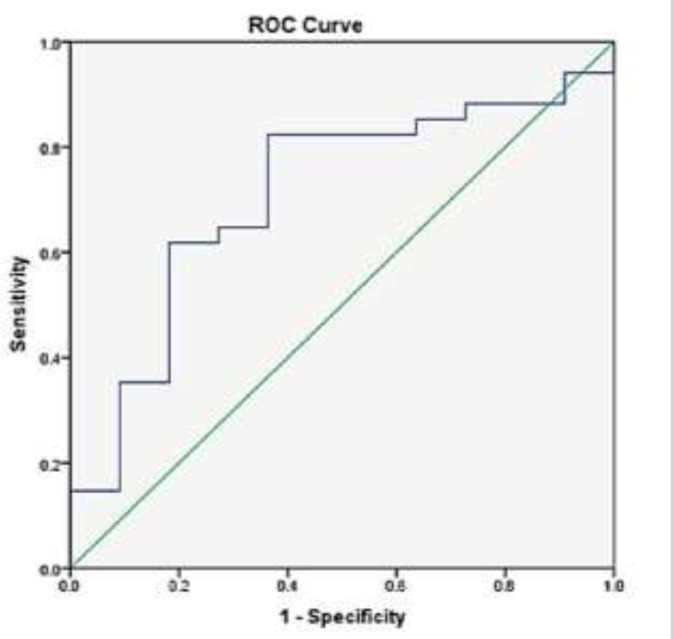
ROC curve for determining the GR-α expression cutoff value for identifying SLE patients with elevated LAC levels. AUC=0.71; 95% CI: 0.53–0.88, P=0.039

The overall sensitivity and specificity of GRα gene expression for CRP and lupus anticoagulant values in SLE patients. The ROC curve analyses were performed to evaluate if GRα gene expression can cause molecular discrimination of SLE patients with and without altered CRP and lupus anticoagulant levels. As presented in fig. 3, the optimal cut-off values of GRα expression levels to differentiate SLE patients with altered CRP levels from normal ones was 290.58, with a sensitivity of 70%, specificity 68%, and area under the curve = 0.70. Moreover, ROC analyses revealed a cut-off value of 160.75 with 82% and 64% sensitivity and specificity, respectively, for the GRα expression levels and area under the curve = 0.71 for the positive lupus anticoagulant test (figure 4). 

**Table 1:** The correlation between GRα gene expression and the laboratory clinical feature of SLE patients

**Clinicopathologic symptoms**	**Median GRα expression levels**	***P*** **-value**
**Oral ulcer**		
Positive (n = 16)	289.4	0.08
Negative (n = 29)	285.8	
**Butterfly rash**		
Positive (n = 21)	269.1	0.45
Negative (n = 24)	405.3	
**Hair loss (alopecia)**		
Positive (n = 17)	275.4	0.88
Negative (n = 28)	321	
**Fever (>37.5 ** ^0C^ ** axillary)**		
Positive (n = 7)	275.4	0.7
Negative (n = 38)	290.5	
**Headache**		
Positive (n = 2)	266.4	0.76
Negative (n = 43)	295.3	
**CVA**		
Positive (n = 1)	1373.7417	0.13
Negative (n = 44)	280.6	
**Vasculitis**		
Positive (n = 5)	263.6	0.87
Negative (n = 40)	290.5	
**Arthritis**		
Positive (n = 32)	290.5	0.96
Negative (n = 13)	275.4	
**Myositis**		
Positive (n = 6)	316.3	0.85
Negative (n = 39)	285.8	
**Serositis**		
Positive (n = 9)	263.6	0.79
Negative (n = 36)	290.5	

CVA: cerebrovascular accident

**Table 2:** Correlation between GRα gene expression and the laboratory parameters of SLE patients

**Laboratory parameters**	**Median GRα expression levels**	***P*** **-value**
**ANA**		
Positive (n = 41)	275.4	0.577
Negative (n = 2)	372.7	
**Anti-dsDNA**		
Positive (n = 39)	275.4	0.503
Negative (n = 6)	515.5	
**Anti-Sm**		
Positive (n = 9)	507.3	0.104
Negative (n = 36)	265.5	
**Anti-cardiolipin antibody**		
Positive (n = 14)	332.3	0.590
Negative (n = 31)	285.8	
**Anti-β2 glycoprotein antibody**		
Positive (n = 13)	269.1	0.689
Negative (n = 32)	290.5	
**Lupus anticoagulant**		
Positive (n = 11)	150.7	0.039
Negative (n = 34)	356.8	
**Leukopenia **(WBC<4000x10^9^/L)		
Positive (n = 18)	272.2	0.694
Negative (n = 27)	356.2	
**Lymphopenia **(1500 x10^9^/L)		
Positive (n = 18)	373.3	0.266
Negative (n = 27)	267.4	
**Thrombocytopenia **(<100,000x10^9^/L)		
Positive (n = 10)	280.6	0.925
Negative (n = 35)	295.3	
**Anemia **(hemoglobin<12 g/L)		
Positive (n = 24)	330.4	0.179
Negative (n = 21)	267.4	
**Decreased GFR **(<60 CC/min)		
Positive (n = 8)	289.4	0.694
Negative (n = 37)	285.8	
Elevated ESR*		
Positive (n = 33)	269.1	0.315
Negative (n = 12)	401.3	
**CRP**		
Positive (n = 20)	405.3	0.032
Negative (n = 25)	180.4	
**C4 deficiency**		
Positive (n = 23)	356.2	0.414
Negative (n = 22)	250.6	
**C3 deficiency**		
Positive (n = 23)	357.3	0.093
Negative (n = 22)	250.6	
**Hematuria**		
Positive (n = 14)	272.2	0.825
Negative (n = 31)	295.3	
Proteinuria**		
Positive (n = 13)	269.1	0.582
Negative (n = 32)	325.7	
**Pyuria**		
Positive (n = 9)	272.2	0.825
Negative (n = 36)	295.3	

*Calculated according to the age of patients

** more than 500mg/24h

GFR=glomerular filtration rate, ESR=erythrocyte sedimentation rate, CRP=c-reactive protein, C4= complement component 4, C3= complement component 3

## Discussion

The For a long time, systemic lupus erythematosus (SLE) pathogenesis was under a magnifying glass, and several mechanisms have been proposed to describe the formation of the disease. Thus far, no medication is effective as glucocorticoids for ameliorating the disease symptoms; however, some patients showed resistance phenotype based on the response rate (23). It has also been reported that the aberrancy in the GRα gene expression and its downregulation may attenuate the glucocorticoid effectiveness and induce glucocorticoid resistance in SLE (16). Given these, in the present study, we aimed to evaluate whether the expression of gene encoding GRα, the dominant form of GRs, could determine the outcome of the newly diagnosed SLE patients. Interestingly, our results showed that comparing with the aged-matched healthy individuals, the expression levels of the GRα gene were reduced in SLE patients before treatment, suggesting that it could reduce the expression of the receptor. We also found that patients with severe disease had lower expression of the GRα gene than those with a more significant activity of the disease. However, the expression of this receptor gene did not have an association with the activity of the disease. In agreement with our results, Du et al. have also reported that the downregulation in the GRα gene expression is associated with the induction of resistance against glucocorticoids (24). In another study conducted on 15 SLE patients, it became evident that the expression of the GRα gene has a lower level as compared to the healthy counterparts due to the hypermethylation of GRα promoter (25). Li et al. have indicated that the expression of the GRα gene was significantly downregulated in SLE patients as compared to the control group. However, unlike our results, they also succeeded in finding a correlation between the expression of this receptor and the SLE Disease Activity Index scores (13). It should be noted that perhaps the difference in the sample size and the bodyweight could be the restrictions that explain why we failed to find any correlation between the disease severity, the clinical and the laboratory features of SLE patients, and the GRα gene expression levels.

Among the long list of parameters, we found a significant correlation between the downregulation of the GRα gene and the values of CRP and lupus anticoagulant in SLE patients. As a standard inflammatory marker in SLE patients, so far, numerous studies have evaluated the correlation between the expression levels of the GRα gene and the values of CRP. However, in many cases, there are conflicting results. Manenschijn et al. have indicated that ER22/23EK polymorphism in the GRα gene, associated with glucocorticoid resistance induction, correlates with the reduced CRP levels (26). In contrast, Kuningas et al. have reported that the SLE patients who had such polymorphisms in gene encoding GRα manifested the higher values of CRP (27). In the present study, we reported that those SLE patients with the reduced levels of GRα gene had higher values of CRP which can be a sign of inflammation.

Moreover, we reported that GRα gene expression levels had 70% sensitivity and 68% specificity to predict CRP values in the patients. We also reported for the first time that GRα gene expression levels could have a positive correlation with lupus anticoagulant levels with the sensitivity of 82%. It is well-established that lupus anticoagulant is a significant risk factor for SLE patients' arterial and venous thrombotic complications (28). The results of previous studies have introduced the triangle between lupus anticoagulant, C3, and C4 deficiency as an indicator of thrombosis and obstetric complications in the patients (29-31). In a study conducted by Rasuli-Saravani et al., it has also been reported that there is human leukocyte antigen (HLA) gene complex that could control the lupus anti-coagulant levels in Iranian patients with SLE (32). 

Given this association, it is reasonable to assume that HLA polymorphism could be a reason that we found a positive correlation between downregulation in the GRα gene and the expression of lupus anti-coagulant in sera of patients. This study was not without limitations, due to targeting newly diagnosed patients which is the strong point of the study who did not receive treatment, we could not increase the sample size. Taken together, the results of the present study declared that although it was not significant, GRα gene expression downregulation in SLE patients as compared to the healthy counterparts, we found a negative correlation between the expression of the GRα gene and CRP value and a positive correlation between that gene expression and lupus anticoagulant positivity. Moreover, GRα gene expression did not correlate with lupus disease activity and severity according to SLEDAI2k. However, further studies in this area are required.
